# Interaction between Host Cells and Microbes in Chemotherapy-Induced Mucositis

**DOI:** 10.3390/nu5051488

**Published:** 2013-04-29

**Authors:** Andrea M. Stringer

**Affiliations:** School of Pharmacy and Medical Sciences, University of South Australia, Adelaide 5001, Australia; E-Mail: andrea.stringer@unisa.edu.au; Tel.: +61-8-8302-1760; Fax: +61-8-8302-2389

**Keywords:** intestine, microbiota, chemotherapy, mucositis, toll like receptors, mitogen activated protein kinase

## Abstract

Cancer patients receiving chemotherapy often develop mucositis as a direct result of their treatment*.* Recently, the intestinal microbiota has attracted significant attention in the investigation of the pathobiology of mucositis, with a number of studies investigating the effects of chemotherapeutic agents on the microbiota. With significant effects on the intestinal microbiota occurring following the administration of chemotherapy, there is now interest surrounding the downstream pathological effects that may be associated with the altered intestinal ecology. This review seeks to identify links between signalling pathways previously demonstrated to have a role in the development of mucositis, and the altered intestinal microbiota.

## 1. Introduction

Cancer patients receiving chemotherapy often develop mucositis as a direct result of their treatment. It is a major oncological problem reported in approximately 40% of patients undergoing standard dose chemotherapy and in almost all patients receiving high dose chemotherapy and stem cell transplantation. This condition was originally thought to be limited to the mucous membranes throughout the alimentary tract, although more recent research has indicated that other tissue layers and the luminal contents and its inhabitants are likely to be involved [1]. The entire alimentary tract (mouth to anus) is affected. Clinical symptoms of intestinal mucositis are the result of ulceration, and include abdominal pain, nausea, vomiting, bloating, diarrhoea, constipation, and subsequent weight loss [1]. 

## 2. Pathobiology of Mucositis

Cytotoxic chemotherapy is a common treatment for malignancies which has been in use for approximately 50 years [2]. It can cause functional and structural changes to the gastrointestinal tract (GIT) [3]. Common gastrointestinal symptoms following chemotherapy include heartburn, abdominal pain, diarrhoea (and constipation), bloating and nausea [4]. These symptoms arise as the result of the damage caused by chemotherapy agents [5]. Abdominal pain is caused by the extensive damage occurring in the small intestine. Diarrhoea and constipation are thought to be caused by the alteration in absorptive functions of cells, goblet cell and mucin distribution and composition, and bacterial interactions with these cells and metabolites of the drugs themselves [6,7]. Cytotoxic drugs are known to act by inducing apoptosis in cancer; apoptosis is also induced in the GIT [8,9,10]. There are limited ways the mucosa and underlying layers of the GIT can respond to damage. These are the same ways that chemotherapy causes damage to the GIT, including cell death, which leads to villous atrophy and crypt ablation in the small intestine, and crypt ablation in the large intestine [4].

The currently accepted hypothesis for the development of alimentary mucositis (AM) suggests that there are five intertwined phases, namely; (1) initiation; (2) up-regulation and generation of messenger signals; (3) signal amplification; (4) ulceration and (5) healing [11,12,13]. This current hypothesis has been supported in both animal and human studies [14,15,16,17,18,19]. Of particular interest is the ulceration phase, where pathological evidence of mucositis is observed, both macroscopically (ulcers) and microscopically (cell changes). Some of these changes observed in animal models and in humans include histological damage [1,10,16,20] and mucin secretion during mucositis [16,21,22,23].

## 3. Possible Roles for Microbiota in the Pathobiology of Chemotherapy-Induced Mucositis

Recently, the microbiota has attracted significant attention in the investigation of the pathobiology of mucositis. The role of the microbiota in the development of chemotherapy induced mucositis has been investigated on a number of levels, including roles in the above-mentioned 5-phase model of mucositis developed by Sonis [12,13,24]. This model is the benchmark model for the pathobiology of mucositis and is a benchmark for current investigative studies in the field. Breaking this model down, roles for microbiota and associated host-microbe responses can be attributed to the different phases of the model.

### 3.1. Initiation and Generation of Reactive Oxygen Species

Chemotherapy treatment causes damage to all tissue types in the gastrointestinal tract. Chemotherapy agents act on and damage crypt cells directly by causing DNA damage or indirectly through production of reactive oxygen species (ROS) (free radicals) and ceramide synthesis [12,13,24]. Reduction of oxygen via univalent reduction results in the formation of superoxide radical, followed by hydrogen peroxide [25]. Superoxide radical is impermeative, however hydrogen peroxide is isoelectric with water and can diffuse freely across the cell membrane. In the intestine, both host and bacterial cells usually contain enzymes to protect vital biological molecules (such as DNA, proteins, and lipids) against attack from ROS [25]. Intestinal oxygenation is affected by the production of ROS, and this in turn will have effects on the bacterial symbionts present in the intestine [25]. Recent studies have suggested that the environmental conditions (e.g., oxygen levels) play a role in determining the composition of intestinal bacterial communities [26]. Inversions of bacterial communities have been observed in patients with ileostomies, with increases seen in Lactobacilli and Enterobacteriaceae (aerotolerant bacteria) at the expense of anaerobes [26]. Normal proportions were restored following closure of the ileostomies, demonstrating the adaptability of the microbiota under the influence of oxygen. 

Under homeostatic conditions, characterised by a state of low-grade inflammation in the intestine, nicotinamide adenine dinucleotide phosphate-oxidase (NADPH) oxidase (NOX)/dual oxidase (DUOX) enzymes appear to be responsible for the production and tight control of ROS production [27,28]. However, with excessive production of free radicals during the initiation phase of chemotherapy-induced mucositis, enzymatic supply responsible for this tight control of ROS may become exhausted, allowing ROS access to these vital molecules, allowing damage to cells, tissues and blood vessels. Of greater concern is the initiation of downstream biological events.

### 3.2. Nuclear Factor Kappa B (NFκB) Up-Regulation, Message Generation, and Amplification

Chemotherapy agents can also activate the transcription factor nuclear factor kappa B (NFκB), both directly and through the generation of ROS, which in turn up-regulates approximately 200 genes, (most notably pro-inflammatory cytokines) often resulting in feedback loops to create more damage [12,13,24]. The intestinal microbiota may play a key role in this phase through the activation of toll like receptor (TLR) signalling [29,30]. The constant residence of microbes in the intestine results in constant exposure to TLR ligands, including peptidoglycans, lipopolysaccharide (LPS) and bacterial DNA. These ligands result in a state of low-grade inflammation [31,32,33], and the bacteria are processed so parts can be transported intracellularly, allowing binding to NOD receptors. Activation of NOD receptors is believed to modulate the inflammatory response activated by TLR activation [34].

The most likely TLRs involved in intestinal mucositis are TLR2, TLR4, TLR5 and TLR9. Once activated by intestinal microbiota, TLRs can also up-regulate NFκB through complex signalling pathways [35], further compounding the inflammatory response through the generation and amplification of tumour necrosis factor (TNF)-α, IL-1β, and IL-6 [13]. Further highlighting the importance of microbial balance in the intestine is the evidence that commensal microbiota can also decrease NFκB activation [36,37]. Commensal species of bacteria, *Bifidobacterium infantis* and *Bacteroides thetaiotaomicron* have both been shown to decrease NFκB activation, with subsequent decreases in endotoxin levels, and plasma pro-inflammatory cytokine levels [36,37,38]. Another commensal bacterium thought to decrease NFκB activation is *Faecalibacterium prausnitzii* [39]. *F. prausnitzii* is able to secrete a substance inducing the production of IL-10, an anti-inflammatory cytokine, resulting in decreased NFκB activation and attenuated inflammation [39]. These findings may suggest that careful regulation of the commensal microbiota following chemotherapy may assist in sustaining intestinal structural integrity through a reduced inflammatory response. However, further investigation into the role of the commensal microbiota in maintaining intestinal structural integrity is required to confirm this hypothesis.

## 4. Mucous Mechanisms of Modulating Microbiota

Intestinal mucins are high molecular weight glycoproteins secreted by goblet cells. Previous studies have demonstrated significant effects on intestinal mucins. Mucins play a key role in protecting the integrity of both the intestinal tissue, and the intestinal microbiota [40]. Their structure provides attachment sites and nutrients for microbes, whilst simultaneously protecting the mucosa from bacterial overgrowth and/or penetration [41]. Another key role is to protect the epithelium from digestion by bacterial enzymes, by acting as substrates for enzymes such as α-galactosidase, β-*N*-acetylgalactosaminidase, sialidase, β-glucuronidase, blood group degrading enzymes, and proteases [41].

Goblet cells rapidly discharge most or all of their stored intracellular mucin in response to a variety of stimuli, including cholinergic stimuli, intestinal anaphylaxis, and chemical or physical irritation [41]. The differential secretion of mucins from surface epithelium and glands may provide a mechanism for the modulation of the protective mucous layer in response to acid secretion, or the presence of bacteria or noxious agents in the lumen [42]. Interleukin (IL)-1, IL-4, IL-6, IL-9, and tumor necrosis factor alpha (TNF)-α have been shown to stimulate the rapid release of mucin from cells [43]. Other bioactive factors from intestinal bacteria or host immune cells may also affect the goblet cell dynamics and mucous layer. Further intertwined involvement of the intestinal microbiota, mucins and interleukins has also been demonstrated by a number of other earlier studies [43,44,45,46]. In the same animal model of mucositis, the intestinal microbiota has been shown to be significantly altered, with decreases in commensal bacteria and increases in potential pathogens [47]. This has been shown to occur at similar times during the development of mucositis to significant increases in the cavitation of goblet cells in the colon [23]. Intertwining with these events is increases in both tissue levels and circulating serum levels of TNF, IL-1β, and IL-6 [19,20].

## 5. Effects of Chemotherapy

### 5.1. Effects of Chemotherapy on the Intestinal Microbiota

Several studies have demonstrated the effects of chemotherapy agents on the intestinal microbiota [48]. One of the most investigated agents is irinotecan, due to the involvement of intestinal microbiota in its metabolism [6]. Irinotecan (used to treat a variety of solid tumours) is a topoisomerase-I inhibitor, converted to active and toxic metabolite SN-38, before being further metabolised to non-toxic metabolite SN-38 glucuronide (SN-38G) [6]. This molecule is excreted into the gastrointestinal tract where it becomes susceptible to processing by bacterial enzymes. Intestinal microbes produce the enzyme β-glucuronidase, which can cleave the glucuronide molecule from the less toxic metabolite of irinotecan, rendering it re-activated and toxic [6]. The effects on the intestinal microbiota caused by irinotecan have been considered important due to this involvement in the drug’s metabolism and potentially compounded toxicities. Studies using single dose (200 mg/kg) irinotecan in Dark Agouti (DA) rats and quantitative 16S rRNA real time PCR have shown significant (*p* < 0.05) increases in *E. coli* (a β-glucuronidase producing bacterium) following its administration, with significant decreases (*p* < 0.05) in *Bifidobacterium* spp. and *Lactobacillus* spp., which do not produce β-glucuronidase [14,21,47]. These changes were also associated with an increased staining intensity for β-glucuronidase in the intestine [47]. A more recent study by Lin and colleagues in 2012 has also demonstrated variations in intestinal microbes using molecular techniques [49]. Irinotecan (3 × 125 mg/kg) was shown to increase the *Clostridium* cluster XI and *Enterobacteriacaea* groups of bacteria, both of which harbour opportunistic pathogens. In addition, studies looking at gene expression analysis using microarray techniques have also shown members of the TLR signalling pathway to be overwhelmingly upregulated [50,51]. These findings may suggest that TLR signalling contributes to mucositis (a multifactorial process [13]), as TLR signalling is has been shown to be upregulated in response to increased bacterial ligands, resulting in further downstream upregulation of NFκB and pro-inflammatory cytokines [35].

Antimetabolite 5-Fluorouracil (5-FU), used to treat colorectal, breast and liver cancers, has been shown to be associated with changes to the intestinal microbiota. A study by von Bultzingslowen and colleagues in 2003 demonstrated that whilst there were no significant changes to levels of anaerobic bacteria in the small or large intestine following 5-FU administration (6 × 50 mg/kg) in Lewis rats, there was a shift in composition from predominantly Gram positive to Gram negative bacteria [52]. A later study by Stringer and colleagues in 2009 showed no significant findings, however peaks were observed following 5-FU administration (150 mg/kg, single dose) in *E. coli* at 48 h, *Clostridium* spp*.* at multiple time points, and *Staphylococcus* spp*.* at 24 h. Decreases were observed in *Lactobacillus* spp. at 24 h and *Enterococcus* spp. at 2 h–48 h following treatment [22]. A combined 5-FU/Irinotecan regimen (irinotecan 50 mg/kg + 5-FU 50 mg/kg administered weekly for two weeks) elevated *Clostridium* cluster XI and *Enterobacteriacaea*, but to a lesser extent than the irinotecan regimen [49].

In clinical studies, changes to intestinal microbiota have also been demonstrated. In a study by van Vliet and colleagues in 2009, paediatric acute myeloid leukaemia patients receiving AML-97 (*i.e.*, high-dose cytarabine, daunorubicine, and etoposide [ADE I and ADE II]; amsacrine, high-dose cytarabine, and etoposide [MACE]; and mitoxantrone and high-dose cytarabine [MidAC]) were shown to have decreased intestinal microbial diversity using denaturing gradient gel electrophoresis (DGGE) [29]. Quantitative fluorescent *in situ* hybridisation (FISH) techniques were used to demonstrate significant decreases in total bacteria (*p* < 0.001), *Bacteroides* spp., *Clostridium* cluster XIVa, *Faecalibacterium prausnitzii*, and *Bifidobacterium* spp. (*p* < 0.004 for all) at both early and late time points following each cycle of chemotherapy [29]. Recovery was observed for both *Clostridium* XIVa and *F. prausnitzii* at 6 weeks following treatment. However, *Bacteroides* spp*.* and *Bifidobacterium* spp*.* were still lower in the end-of-treatment samples compared with the healthy controls [29]. This suggests that the intestinal microbiota may have the potential to be restored following chemotherapy, and the possibility of manipulation of the microbiota to promote healing following mucositis is an area requiring investigation. A study by Zwielehner and colleagues in 2011 using molecular techniques to quantify bacteria in patients receiving varied chemotherapy regimens has shown a significant decrease in total microbiota (*p* = 0.037), similar to the van Vliet study in 2009. Similar decreases were also observed in *Bifidobacterium* spp., *Lactobacillus* and *Clostridium* cluster IV. *Faecalibacterium prausnitzii* also decreased dramatically, from 9% to zero following chemotherapy [53]. This study also demonstrated the appearance after chemotherapy of undetected bacteria prior to chemotherapy, which could suggest a potential risk to the patient of infection from these species. 

### 5.2. Effects of Chemotherapy on Mucins

Mucins have been shown to be associated with mucositis [22,23,54,55,56]. Dark Agouti (DA) rat models of mucositis have been used to determine a significant association with mucin secretion and expression [22,23]. Cavitated goblet cells (goblet cells which have released mucins through exocytosis) have been shown to significantly increase (*p* < 0.05) from 96 h to 144 h following irinotecan (200 mg/kg, single dose) treatment. Muc4 expression has also been shown to increase significantly (*p* < 0.05) in the jejunum compared to controls [23], which may suggest accelerated trans-membrane mucus production.

In a study investigating 5-FU-induced mucositis (150 mg/kg, single dose), goblet cell numbers decreased significantly in the jejunum (*p* < 0.05), and the percentage of cavitated goblet cells increased significantly (*p* < 0.05) from 24 h to 72 h after treatment compared with controls [22]. Furthermore, a Wistar rat model of 5-FU-induced mucositis (5 × 50 mg/kg) caused a significant (*p* < 0.05) decrease in mucin levels compared to controls in the jejunum and colon [55], suggesting mucin expression was decreased. Mucin expression has also been shown to be significantly (*p* < 0.05) decreased in a Wag/Rij rat model of methotrexate (MTX) induced mucositis (2 × 10 mg/kg), with Muc 2 expression decreased significantly from 72 h to 44 h following MTX administration [56]. The findings of these studies suggest that mucins are released following chemotherapy, which may be due to effects of IL-1, 4, 6 and 9, and TNF-α, also upregulated following chemotherapy.

### 5.3. Effects of Chemotherapy on Pro-Inflammatory Cytokines

Pro-inflammatory cytokines have long been held responsible for the tissue damage associated with mucositis [12,13,15,19,20,24,57]. Studies have shown significant increases in the expression of pro-inflammatory cytokines, with the key offenders TNF, IL-1β [13,19,20,57,58,59] IL-4 [60] and IL-6 [19,20,43,57,58]. In timeline studies of irinotecan by Logan and colleagues, both intestinal and serum levels of NFκB were significantly increased at 6 h–12 h following administration of irinotecan (*p* < 0.05). In the same study, intestinal and serum levels of TNF, IL-1β and IL-6 were also shown to be significantly increased at 6 h–24 h following irinotecan administration compared to controls (*p* < 0.05). The up-regulation of these cytokines is likely to result in the release of mucins from goblet cells, which may have further downstream effects on the intestinal microbiota, due to the subsequently altered environmental conditions in the intestinal mucosa and lumen. 

## 6. Relationships between Intestinal Microbiota, Mucins and Pro-Inflammatory Cytokines

**Figure 1:**
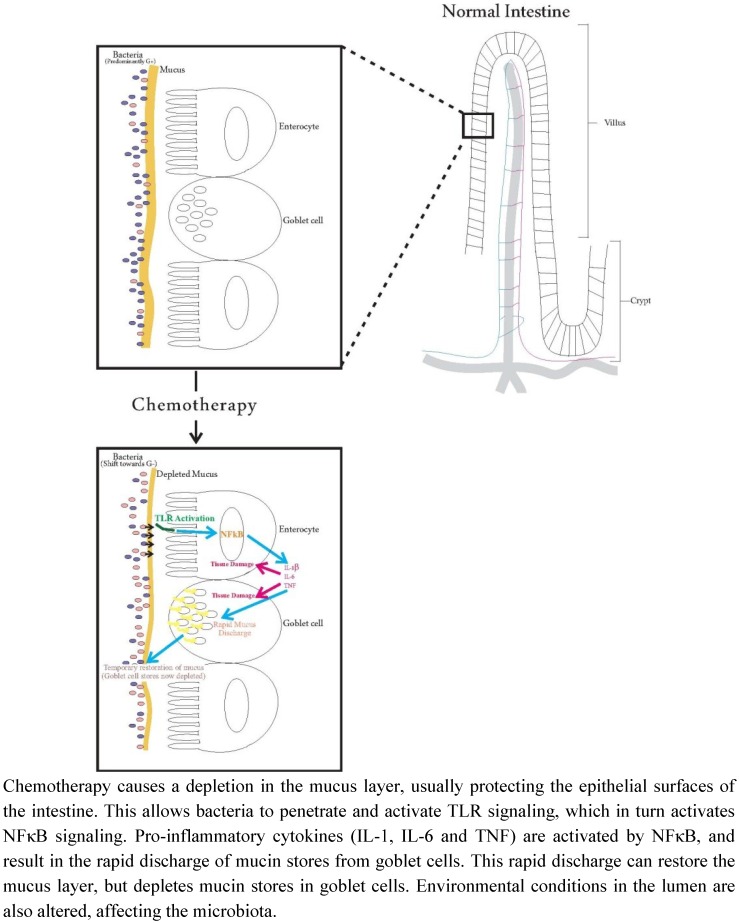
Relationships between mucins, microbiota and cytokines: the effects of chemotherapy.

The timing of events in studies by Stringer and colleagues [23,47], and Logan and colleagues [19,20], supports the notion that these manifestations are connected. These studies have shown that percentage of cavitated goblet cells increased significantly at 24 h–72 h in the colon following irinotecan administration in the same animal model as the Logan studies [19,20,23]. They also showed a significant decrease in the expression of MUC2 at 24 h–72 h. In another study in the same animal model, changes to intestinal microbiota were observed, with a significant increase (*p* < 0.05) in *E. coli* at 24 h–48 h, and a significant decrease (*p* < 0.05) in *Lactobacillus* spp. at 12 h–48 h [47]. When these findings are combined, they suggest that pro-inflammatory cytokines become activated through the activation of NFκB [13], subsequently feeding back to further amplify NFκB signalling, compounding the effects of the cytokines and subsequently the tissue damage. Intestinal microbiota also play a key role in the activation of cytokines through the up-regulation of TLR signalling (and subsequently NFκB signalling) (Figure 1). Pro-inflammatory cytokines can also result in the rapid discharge of mucin stores in goblet cells [43], which may have led to the increased cavitation of goblet cells (Figure 1). The depletion of mucin stores in goblet cells will lead to subsequent effects on the mucus layer in the intestine, which in turn will affect the intestinal microbial ecology by reducing attachment sites and available nutrients (reducing the number of commensal bacteria, such as *Lactobacillus* spp., and allowing other aerotolerant species, such as *E. coli*, the capacity to proliferate) (Figure 1). These events are likely to be overarched by the increases in ROS and its downstream effects initiated soon after chemotherapy is administered.

## 7. Future Directions

Further investigations are required to fully elicit the intricate role of intestinal microbes in the development of gastrointestinal mucositis. Studies investigating the effects of ROS produced following administration of chemotherapy and the effects on microbiota, and investigating the intricate relationship between mucins and microbes in the development of mucositis following administration of chemotherapy agents should be carried out. The findings from such studies are likely to provide the level of evidence required to suggest some possible intervention targets to improve patient outcomes following cancer therapy.

## 8. Conclusions

Chemotherapy-induced mucositis is a complex process involving a number of tissue components and a moiety of signalling pathways. Intestinal microbiota has been shown to be altered following chemotherapy, and may therefore play a key role in the development of mucositis. Consequently, the intestinal microbiota, or components of the signalling systems activated by it, may be considered as potential targets for therapeutic intervention.
